# Central retinal artery occlusion and cerebral infarction associated with Mycoplasma pneumonia infection in children

**DOI:** 10.1186/s12887-016-0750-3

**Published:** 2016-12-09

**Authors:** Yunguang Bao, Xiaobing Li, Kaixuan Wang, Chan Zhao, Xiumei Ji, Mizu Jiang

**Affiliations:** 1 Department of Pediatrics, Jinhua Hospital of Zhejiang University, Jinhua Municipal Central Hospital, Jinhua, 321000 Zhejiang Province China; 2 Children’s Hospital, Zhejiang University School of Medicine, Hangzhou, 310003 Zhejiang Province China

**Keywords:** Mycopasma pneumonia, Central retinal artery occlusion, Brain infarction, Pneumonia, Children

## Abstract

**Background:**

Central retinal artery occlusion (CRAO) is an arterial ischemic stroke, rarely occurred in children accompanied with asymptomatic cerebral infarction and almost never involved in severe pneumonia related to Mycoplasma pneumonia infection.

**Case presentation:**

An 8-year-old boy with severe pneumonia related to Mycoplasma pneumonia infection that developed loss of vision in the left eye on the 14^th^ day. No light perception and no pupillary reaction to light were found in the left eye. The fundus examination revealed a cherry red spot with severe retinal edema at the macular and peripapillary area, and the optic disc was pale in the left eye but normal in the right eye, suggesting CRAO in the left eye. No obvious neurological symptoms and signs were observed on presentation. Magnetic resonance imaging of the brain showed an abnormal signal of the left lentiform nucleus, caudate nucleus and within the temporal lobe, suggesting an acute cerebral infarction. The analysis of cerebrospinal fluid showed an increasing leukocyte count, but no any pathogenic microorganisms were found. His respiratory symptoms disappeared promptly after therapy, and the patient was discharged after 11 days later, but there was no light in the left eye 2 months after discharge.

**Conclusion:**

M. pneumoniae infection could be developed the risk for cerebral ischemic stroke, including CRAO in children with severe pneumonia. CRAO is a devastating ophthalmologic event leading to a severe impairment of vision. Patients treated within about 6 h of vision loss had a better visual outcome after the onset of vision loss.

## Background

Childhood stroke is a cerebrovascular event that occurs between 30 days and 18 years of age. The annual incidence of stroke in children is estimated to be 2–4/100,000 in the United States. Ischemic stroke is more common than hemorrhagic stroke [1]. Furthermore, infectious diseases typically precede a significant proportion of acute ischemic strokes (AIS) in children [2]. The most common infectious agent is Varicella zoster virus, followed by Mycoplasma pneumoniae (M. pneumoniae), Chlamydia pneumonia, Parvovirus B19, influenza A virus, and mumps virus infection. These microorganisms have been identified as potential risk factors for arterial ischemic stroke during childhood [1–3]. M. pneumoniae may cause a vascular occlusion type with an extrapulmonary manifestation through local vascular injury in the absence of a systemic hypercoagulable state [4]. In the present case, the patient presented with central retinal artery occlusion (CRAO) in the left eye and cerebral infarction in the left lentiform nucleus and caudate nucleus on the 14^th^ day after onset of the M. pneumoniae respiratory infection. This study was approved by the ethical committee at Jinhua Municipal Central Hospital and Children’s Hospital, Zhejiang University School of Medicine, China. Consent was obtained from their parents.

## Case presentation

A previously healthy 8-year-old boy with a fever and cough lasting for 1 week was admitted to the Department of Pediatrics in Jinhua Hospital of Zhejiang University. Four days prior to admission, the symptoms did not improve after intravenous antibiotic treatment at a local clinic, but more detailed medical history was unavailable. The patient reported no history of cardiovascular disease, coagulation disorders or any other systemic immune disorder, nor was there any recent history of trauma. The patient’s family history was unremarkable. He was alert on admission. His weight was 24 kg, his body temperature was 37.8° Celsius, his pulse rate was 112/min, his respiratory rate was 24/min and his blood pressure was 108/65 mmHg. A physical examination revealed mild congestion of his throat and moderate enlargement of his tonsils. A chest examination revealed coarse breathing sounds and decreased sounds on the right side without any respiratory distress. No crackles were heard in the lung. The neurological examination and the rest of the systemic physical examination were normal.

**Fig. 1:**
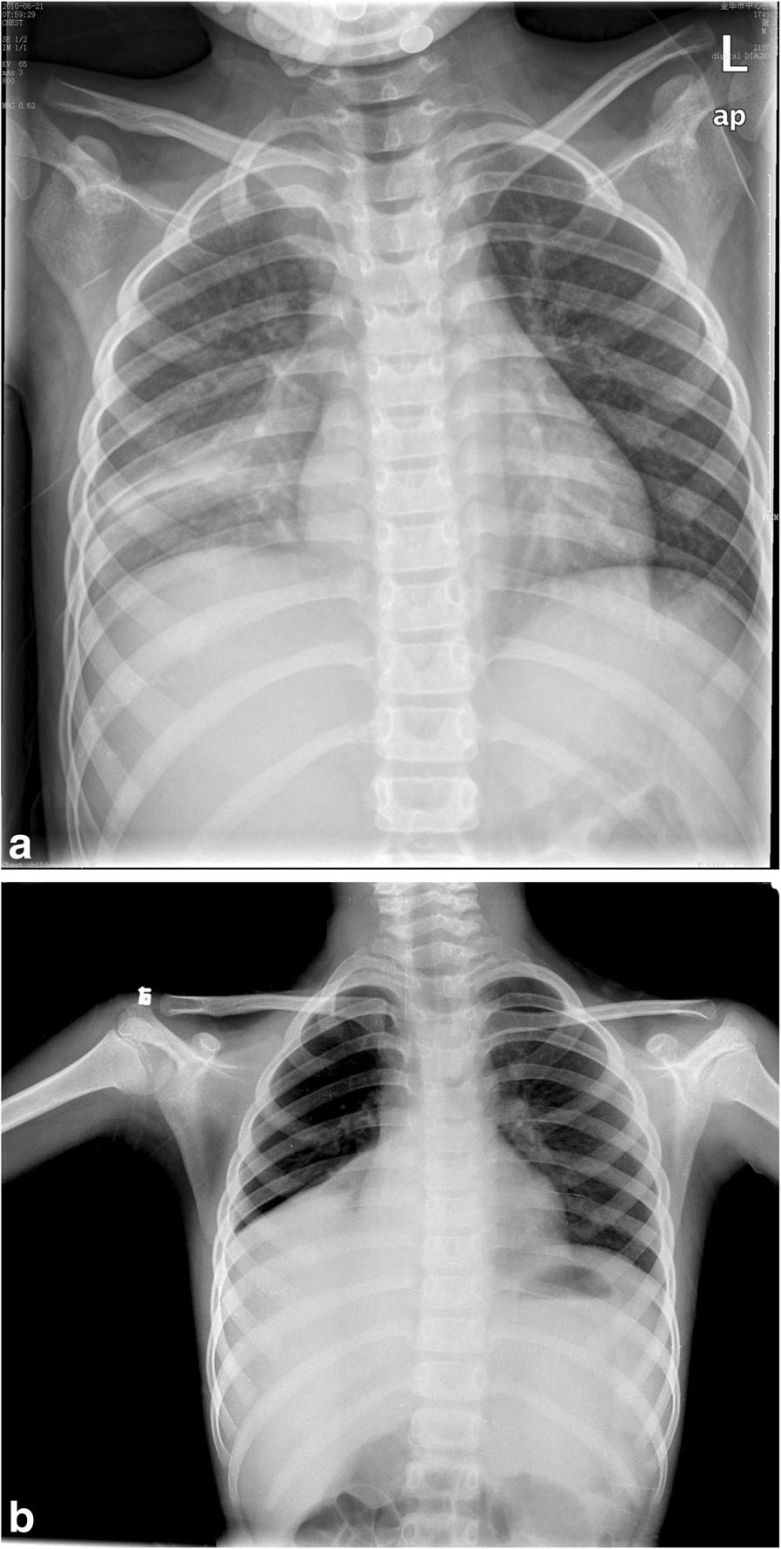
**a** Chest X-ray showed that two lung markings were increased, the high density lower right lung patchy shadows and a small right-sided pleural effusion at the initial presentation. **b** Chest X-ray showed that two lung textures were increased, the right lower lung had a high patchy density, and its edge was smooth. The right rib diaphragm angle was lost. The heart shadow had no obvious increase, and the left diaphragm was normal on the second day after transfer

**Fig. 2:**
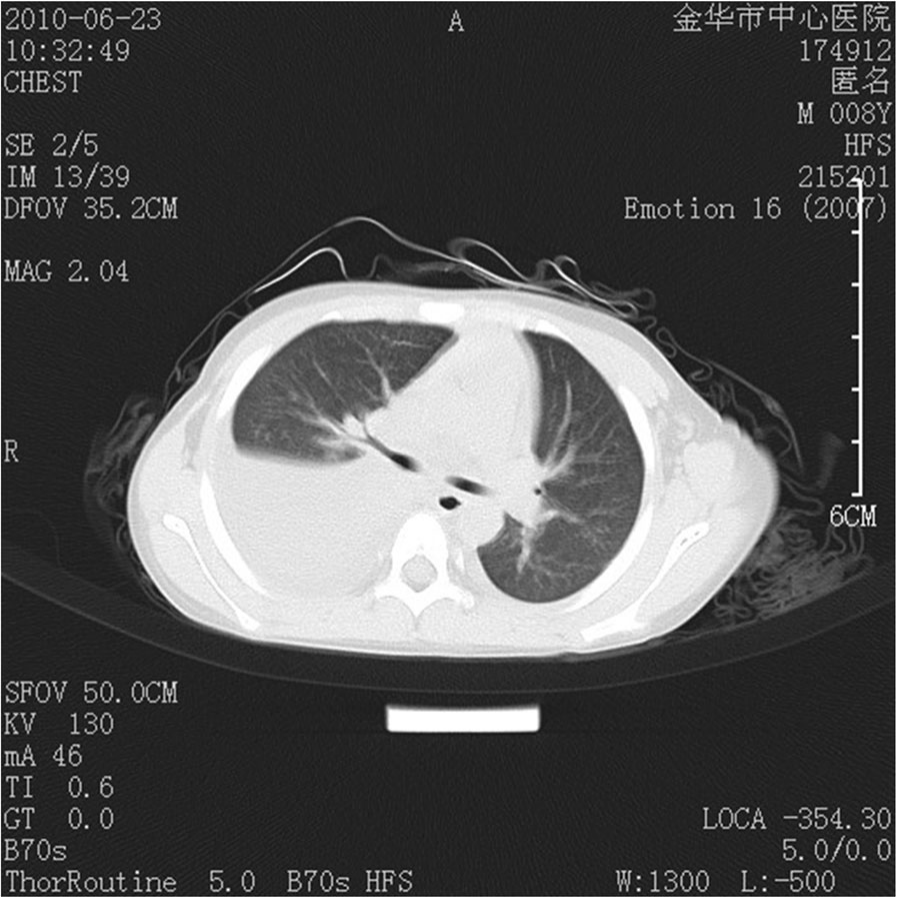
Chest CT: Large high-intensity lesions in his right lower lung lobe, the stenosis of the lower right bronchial lumen and right-sided pleural effusion at the initial presentation

**Fig. 3:**
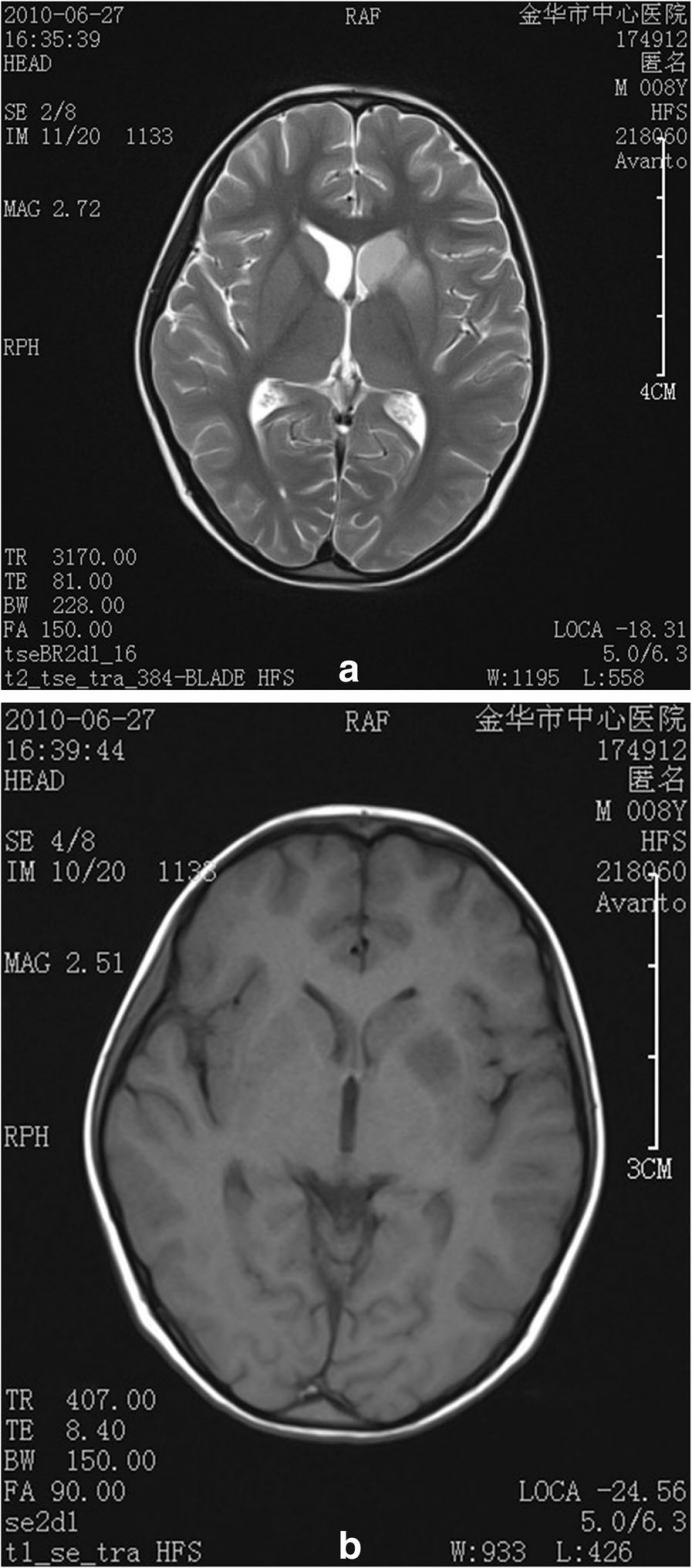
**a**-**b** Magnetic resonance imaging (MRI) of the brain showed a T2W1-weighted high-intensity signal (**a**) and a T1W1-weighted low-intensity signal of the left lentiform nucleus, caudate nucleus and temporal lobe (**b**), suggesting an acute infarction of the brain, including the above regions the first day after transfer

A laboratory investigation revealed a white blood cell count of 9900/cubic mm^3^, with 79.3% neutrophils and 10.2% lymphocytes as well as hemoglobin concentration of 11.3 g/dl and a platelet count of 476,000/cubic mm^3^. The serum biochemistry and lipid profiles were all within normal ranges. The erythrocyte sedimentation rate (ESR) was 22 mm/hr, and C-reactive protein (CRP) was 44.2 mg/dl (normal <8 mg/dl). Antibody titers of IgM to M. pneumonia measured by enzyme-linked immunosorbent assay (ELISA) were negative on admission. The chest radiograph demonstrated that there was a pneumonia patch over his right *lower* lung lobe and a small right-sided pleural effusion (Fig. 1a). The patient was considered to have severe pneumonia, probably due to M. pneumonia, and was given intravenous azithromycin, cefotaxime, ambroxol hydrochloride and dexamethasone. The cough began to improve, but the high body temperature persisted. A computed tomography (CT) on the third day of hospitalization showed a large high-intensity lesion in his right lower lung lobe, stenosis of the lower right bronchial lumen and right-sided pleural effusion (Fig. 2). A chest ultrasound examination on the 4^th^ day of hospitalization showed a bilateral pleural effusion. On the 6^th^ day of hospitalization, the patient cried suddenly and shook his right upper limb after intravenous ambroxol hydrochloride for 10 min. Ambroxol hydrochloride was stopped immediately and changed to azithromycin because of the possibility of adverse reaction to a drug. The shaking of his right upper limb improved, but he was still crying and complained of blurred vision in his left eye. After stopping the intravenous azithromycin because of the possibility of allergic reaction of azithromycin and shifting to water soluble vitamins, the patient was quiet. The physical examination revealed a garden bilateral pupil with an equal 3-mm diameter, and reaction to light was normal at the initial presentation. No headache, no vomiting, no talking disorders, no disturbance of consciousness, no twitching, no convulsions and no limb movement disorders occurred. The neurological examination showed normal muscle strength and muscle tone of limbs, but no pathologic reflex and no other abnormal signs on presentation. On the following day, the patient still complained of loss of vision in the left eye. The ophthalmologic examination showed no light perception and no pupillary reaction to light, but normal anterior segment and intraocular pressure in the left eye. Visual acuity was 1.0 in the right eye. The fundus examination revealed a cherry red spot with severe retinal edema at the macular and peripapillary area, and the optic disc was pale in the left eye, but the examination was normal in the right eye, suggesting central retinal artery occlusion in the left eye. The detailed neurological examination was perforemed as follows: the nasolabial fold was symmetric, and the tongue was midline protrusion; There was no deviation of the mandible, no involuntary movement; Limb muscle strength was grade IV, and muscular tension was normal; Knee reflex and abdominal reflex was normal, and pathological reflex were negative. Initially, he was given oxygen and a low molecular dextran treatment and carteolol hydrochloride to lower the intraocular pressure 29 h after the onset of vision complaint. Electrocardiogram, echocardiography, and Doppler ultrasound of the bilateral carotid artery and vertebral artery were all normal. Magnetic resonance imaging (MRI) of the brain showed an abnormal signal of the left lentiform nucleus, caudate nucleus and within the temporal lobe, suggesting an acute infarction of the brain including the above regions (Fig. 3a–b). He was prescribed tienam to escalate the anti-infection treatment, and early rehabilitation therapy with neurosupplement treatment was initiated, and prepared to transfer the patient to higher level hospital.

One day after the brain infarction, the patient was transferred to Children’s Hospital of Zhejiang University School of Medicine. Laboratory investigations revealed a normal serum coagulation test. The ESR was 105 mm/hr, and CRP was 106.0 mg/dl. The serum titer of the M. pneumoniae particle agglutination test (IgG and IgM) was more than 1:320 (normal <1:80). The analysis of cerebrospinal fluid (CSF) showed a leukocyte count of 40/cumm, with 85% mononuclear cells and 15% multinucleated cells as well as a [Cl^−^] of 112.6 mmol/L and initial positive qualitative protein. The test of the acid-fast bacilli of the CSF and Cryptococcus neoformans were negative. Polymerase chain reaction (PCR) for M. pneumoniae of the CSF was negative. The pathogen DNA detection of M. pneumoniae from respiratory secretion was positive (4.2 × 10^5^ copies). The bacteriological cultures of blood, CSF and pleural effusion were sterile. The PPD test on skin was negative. The levels of serologic cytokines, such as interleukin-6 (IL-6) 159.7 (normal 1.7–16.6) and IL-10 7.6 (normal 2.0–4.0), were obviously increased, whereas the levels of IL-2, IL-4, TNF-ɑ, IFN-ɤ were all normal. The ophthalmologic examination showed no light perception, no pupillary reaction to light and a cherry red spot with severe retinal edema in the macular and peripapillary area in the left eye, but no issues in the right eye. A chest X-ray showed a high patchy density over his right lower lung lobe and a large right-sided pleural effusion. The right rib diaphragm angle was lost. The heart shadow had no obvious increase, and the left diaphragm was normal on the second day after transfer (Fig. 1b). On the basis of his recent medical history and diagnostic workups, we concluded that the diagnosis was acute severe pneumonia with pleural effusion, cerebral infarction and CRAO in the left eye associated with M. pneumoniae infection. Therapy with oxygen inhalation, Tazocin (Piperacillin Sodium and Tazobactam Sodium) antibiotics, low molecular dextran treatment, methylprednisone, gamma globulin, mannitol and nicotinic acid was given, and atropine was injected in the left retrobulbar. The body temperature was normal, CSF was also returned to normal on the 6^th^ day and the cough had disappeared on the 11^th^ day after transfer. MRI of the brain indicated an abnormal signal in the left lentiform nucleus and caudate nucleus (patchy long T1 and long T2 signal), suggesting infarction on the 10th day (not shown), and revealed a softening tendency of the lesions on the left basal ganglia on the 16th day after transfer. Ophthalmologic examination showed no light perception and no pupillary reaction to light, but the retinal edema subsided, the retinal artery was sclerosed, the optic disc was pale and the disc margin was slightly fuzzy at that time. During the 18 days of hospitalization in the children’s hospital, his respiratory symptoms totally subsided and he was discharged.

**Fig. 4:**
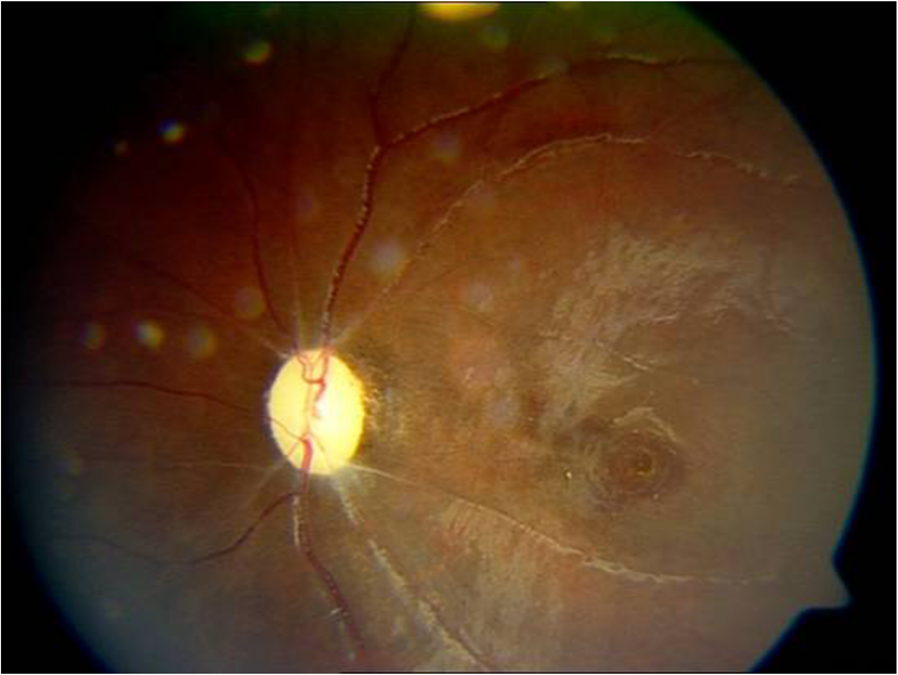
Fundus examination showed that the optic disc on the left eye was clear and pale, C/D = 0.3. The central retinal artery was sclerosed, the retina was normal, but the macular pigment was severely disordered at the 2-month follow-up after CRAO

At the 2 week follow-up, his vision had deteriorated to no light perception OD in the left eye. The retinal cherry red spot had resolved, the disc boundary was clear and pale and the retinal edema had resolved remarkably, but the macular pigment was mildly disordered (not shown). At the 2-month follow-up, there was no light in the left eye, the fundus examination in the left eye revealed the optic disc was clear and pale and the retina was normal, but the macular pigment was severely disordered (Fig. 4). The fundus examination was normal in the right eye.

## Discussion

M. pneumoniae is one of the most common pathogens of respiratory tract infections in children. M. pneumoniae infection has been recognized to be associated with many extra-pulmonary manifestations. including neurologic, cardiac, dermatologic, hematologic, immunological and gastrointestinal complications, among others [5, 6]. The central nervous system is one of the most common sites of infection of M. pneumoniae. They occur in no less than 3 days after onset of M. pneumonia respiratory disease (para-infectious type) and up to 2–3 weeks after respiratory disease subsides (post-infectious type) with a heterogeneous spectrum of clinical manifestations, such as menigoencephalitis, polyradiculitis, acute disseminated encephalomyelopathy, transverse myelitis, aseptic meningitis, seizures, and stroke [3, 6, 7]. The incidence of neurological complications was estimated to range from 1 to 10%, and the mean interval between the onset of respiratory symptoms and neurological manifestations was 9.6 days (range: 2–14 days) [2]. Stroke in children is rare, but it is one of the most severe neurologic complications associated with M. pneumoniae infection, which involves direct central nervous system invasion, immune mechanisms, vascular occlusion, and a hypercoagulable state [4, 6]. CRAO is a devastating ophthalmologic event leading to a severe impairment of vision that presents with acute, painless loss of monocular vision [8]. Frequent etiologies include hypercoagulable states, emboli from cardiac valvular disease, vasculitis, and other risk factors, such as smoking, oral contraception use and vasospasm in migraine histories [9]. To the best of our knowledge, there was no report on the concomitant involvement of both CRAO and cerebral infarction in children associated with M. pneumoniae infection.

To date, 12 children with M. pneumoniae-associated stroke have been reported in the literature [1]. Most patients presented with weakness of one side of the body or face with dysarthria or aphasia. Two cases were found to have arterial occlusion at the level of anterior circulation in the brain and concomitant occlusion of the anterior and posterior cerebral circulation [10]. The CRAO in the left eye that resulted in severe and permanent visual loss, as in this case, has not been reported before. The mechanism of M. pneumoniae infection-associated stroke is still unclear. Thrombosis and hypercoagulable states have been noted in those cases with M. pneumoniae infection. However, the thrombophilia profiles among all 11 patients were normal except for one with a positive anti-cardiolipin IgM antibody [2]. The serum coagulation test in this case was also normal. An immune mechanism had been proposed because of a 2–3 week delay between the occurrence of stroke and the respiratory disease. The immune-mediated response includes autoimmunity, organism-induced immune suppression, immune complex vasculopathy and thrombosis of vessels. In this case, the interval between the onset of respiratory symptoms and the visual loss in the left eye was 14 days. Leonardi et al. proposed that M. pneumoniae infection-related ischemic stroke can be divided into early and late types [3]. Proposed hypotheses include direct invasion of M. pneumoniae occurring in the early type and immune-mediated damage contributing to a 2–3 weeks delay type. Vascular occlusion extrapulmonary manifestations comprise disorders of vascular origin involving both direct and indirect mechanisms. Narita et al. reported elevated levels of IL-6, IL-8, and IL-18 mostly in patients with mycoplasma encephalitis classified as the late-onset type [4]. M. pneumoniae has been isolated from the cerebrovascular fluid of stroke patients [11], and it may induce chemokines, such as TNF-ɑ and IL-8, in the vasculature, resulting in local vasculitic or thrombotic vascular occlusion without a systemic hypercoagulable state (direct mechanism) [4]. We found elevated serologic levels of IL-6 and IL-10 in this case, suggesting that the CRAO could be associated with an immune response induced by M. pneumonia infection.

The management of mycoplasma-associated stroke remains controversial [6, 12]. The therapeutic window for rescuing ischemic but still viable tissue is very short [13]. Neuronal death and retinal infarction evolve progressively in a time-dependent fashion that is determined by both the duration and severity of the ischemic insult. Early reperfusion of ischemic tissue has the potential to limit the cellular, biochemical and metabolic consequences of retinal ischemia that ultimately lead to irreversible vision loss. Retinal artery occlusion is associated with a poor visual prognosis, and aggressive management with ocular massage, anterior chamber paracentesis, and carbogen therapy does not appear to improve the outcome [13, 14]. Animal model studies have suggested that the ideal therapeutic window for CRAO is less than 4 h [15]. However, it is likely that 6 h is acceptable for many patients with incomplete CRAO. Hattenbach et al. showed that patients treated within 6.5 h of vision loss had a better visual outcome than those treated between 6.5 and 12 h after the onset of vision loss [16]. Our patient was diagnosed with CRAO approximately 29 h after the onset of CRAO. The patient was suddenly crying in the infusion process, together with a transient shaking of right upper limb, and a complaint of blurred vision, which should be considered to be cerebral embolism and retinal arterial embolization because of inflammatory embolus. As the child was not clearly described his complaint, and the doctor was short of experience, also the physical examination was not careful especially in the occurrence of vision complaint. Therefore, the diagnosis of this case was initially delayed when the onset of CRAO. In spite of the immunosuppressive therapy, including steroids and intravenous immunoglobulin, as well as other conservative treatments, such as reducing intra-ocular pressure, ocular massage, and vasodilators, the visual prognosis in the left eye is very poor.

## Conclusions

We should be aware of the risk for cerebral ischemic stroke, including CRAO, as a complication during the clinical course of M. pneumoniae respiratory disease in children. CRAO is a devastating ophthalmologic event leading to a severe impairment of vision and should be treated within about 6 h after the onset of vision loss.

## Abbreviations

AIS: Acute ischemic strokes
CRAO: Central retinal artery occlusion
CRP: C-reactive protein
CSF: Cerebrospinal fluid
CT: Computed tomography
ELISA: Enzyme-linked immunosorbent assay
ESR: Erythrocyte sedimentation rate
M. pneumonia: Mycoplasma pneumonia
MRI: Magnetic resonance imaging
PCR: Polymerase chain reaction
